# The Affective Bases of Risk Perception: Negative Feelings and Stress Mediate the Relationship between Mental Imagery and Risk Perception

**DOI:** 10.3389/fpsyg.2016.00932

**Published:** 2016-06-24

**Authors:** Agata Sobkow, Jakub Traczyk, Tomasz Zaleskiewicz

**Affiliations:** ^1^Department of Cognitive and Individual Differences Psychology, Faculty in Wroclaw, SWPS University of Social Sciences and HumanitiesWroclaw, Poland; ^2^Department of Economic Psychology, Faculty in Wroclaw, SWPS University of Social Sciences and HumanitiesWroclaw, Poland

**Keywords:** imagery, risk perception, affect, stress, emotions, blood pressure, risk-as-feelings hypothesis, risk assessment

## Abstract

Recent research has documented that affect plays a crucial role in risk perception. When no information about numerical risk estimates is available (e.g., probability of loss or magnitude of consequences), people may rely on positive and negative affect toward perceived risk. However, determinants of affective reactions to risks are poorly understood. In a series of three experiments, we addressed the question of whether and to what degree mental imagery eliciting negative affect and stress influences risk perception. In each experiment, participants were instructed to visualize consequences of risk taking and to rate riskiness. In Experiment 1, participants who imagined negative risk consequences reported more negative affect and perceived risk as higher compared to the control condition. In Experiment 2, we found that this effect was driven by affect elicited by mental imagery rather than its vividness and intensity. In this study, imagining positive risk consequences led to lower perceived risk than visualizing negative risk consequences. Finally, we tested the hypothesis that negative affect related to higher perceived risk was caused by negative feelings of stress. In Experiment 3, we introduced risk-irrelevant stress to show that participants in the stress condition rated perceived risk as higher in comparison to the control condition. This experiment showed that higher ratings of perceived risk were influenced by psychological stress. Taken together, our results demonstrate that affect-laden mental imagery dramatically changes risk perception through negative affect (i.e., psychological stress).

## Introduction

Almost every day people face dilemmas, in which they have to decide about accepting or rejecting risk. For example, they choose whether to drive unsafely in order not to miss the meeting, whether to have sex with an unknown partner, whether to invest savings in volatile stocks, etc. Theoretical models have postulated that the decision concerning risk acceptance might result from risk perception in a way that increased perceived risk decreases the likelihood of engaging in a risky behavior and vice versa (e.g., Sarin and Weber, 1993). There is empirical evidence supporting these assumptions (Weber and Milliman, 1997; Weber et al., 2002; Blais and Weber, 2006). However, there is also some disagreement concerning the issue of which factors determine risk perception. Normative decision models or axiomatic models of perceived risk assume that risk assessment is based on the calculation of outcomes and their probabilities (Neuman and Politser, 1992; Jia et al., 2008), but in real-life situations quantitative information about probabilities and consequences is rarely available or it is very difficult to process. Therefore, perceived risk and risk-taking behavior often result from people's gut feelings (Bechara et al., 1997; Wagar and Dixon, 2006), past experiences (Traczyk and Zaleskiewicz, 2015), or anticipated emotions (Mellers et al., 1999). People simply feel or intuitively experience the size of a potential danger. The experimental project presented in this paper indeed shows that negative affect and the state of stress resulting from mental imagery might have a meaningful impact on risk perception.

The role of emotions in both risk perception and risk taking has been extensively studied in behavioral decision research (Bechara et al., 1996; Loewenstein et al., 2001; Rottenstreich and Hsee, 2001; Bechara, 2004; Slovic et al., 2007). For instance, Loewenstein and Lerner (2003) argued that a combined effect of anticipatory emotions (i.e., arising from considering consequences) and incidental emotions (i.e., arising from factors unrelated to the decision) influences decision making. More specifically, Loewenstein et al. (2001, 270) postulated in their risk-as-feelings model that “responses to risky situations (including decision making) result in part from direct (i.e., not cortically mediated) emotional influences, including feelings such as worry, fear, dread, or anxiety.” Moreover, they assumed that some emotional factors indirectly influence risky choices with only little or even without cognitive control as in panic reactions to threatening stimuli. The risk-as-feelings hypothesis posits that one of the factors that is responsible for evoking strong risk-related emotions is vividness. In other words, intense feelings associated with risk perception might be produced by vivid mental representations or visualizations of a risky situation. This assertion seems to be supported by findings from cognitive psychology showing that emotions are strongly related to mental imagery (Kosslyn, 1994) and that imagining unpleasant events leads to experiencing more anxiety (Holmes and Mathews, 2005). Results from studies conducted in the so-called psychometric paradigm (see Slovic, 2000, 2013) also revealed a relationship between mental imagery and risk acceptance in ecological and financial domains (Peters and Slovic, 1996; MacGregor et al., 2000; Peters et al., 2006). For example, people who produced more negative images of risk showed a stronger risk-averse bias (Slovic et al., 1991).

A more direct empirical test of the risk-as-feelings hypothesis and its assumption that mental visualizations play an important role in risk perception and risk acceptance has recently been provided by Traczyk et al. (2015). These authors showed in a series of experiments that imagining negative consequences of risk elicits negative affect and feelings of stress, which in turn decrease people's willingness to engage in a risky behavior. The present paper replicates and extends this work by showing that the vividness and intensity of negative risk images influences risk perception and that this relationship is mediated by negative affect associated with feelings of stress. Experiments presented in this article differ from previous research in at least three ways. Firstly, using the experimental design we are now able to directly test causal relationships between imagery, affect and risk perception which was a main objective of Experiment 1. Secondly, we control task-involvement to report that it is negative rather than positive affect that influences risk perception (Experiment 2). Thirdly, we directly verify the hypothesis that changes in risk perception are determined by stress, which does not have to be related to a risky activity (Experiment 3).

The further part of this paper presents methods and results of three experiments. Experiment 1 showed that imagining negative consequences of risk produces negative affect, which, in turn, increases perceived risk. Results of Experiment 2 documented that imagining negative, but not positive, risk consequences is responsible for the increase in perceived risk and that this relation is mediated by negative affect. Finally, Experiment 3 revealed that even incidental stress might have an impact on risk perception.

## Experiment 1

### Method

#### Participants

One hundred and five unpaid volunteers (88 females; mean age = 25.1 years; *SD* = 6.7) participated in this study. Participants provided informed consent before the experiment. The participation in this study was voluntary, anonymous, and in agreement with the guidelines of the Ethical Committee.

#### Procedure

Participants were randomly assigned to either experimental or control conditions. In both groups, participants read brief descriptions of five risky situations (e.g., “Ignoring persistent medical problems”; see Table S1) randomly presented on the computer screen. In the experimental condition, they were asked to imagine and write down three possible negative consequences of each risky situation. In the control condition, participants had to solve three simple arithmetic problems (e.g., “17 − 5 = ?”) instead of visualizing negative risk consequences. Each risky situation was followed by six questions concerning: (1) emotions evoked by negative risk consequences (three questions), (2) risk perception (two questions), and (3) the intensity of mental images of risk (one question; by asking about intensity, we intended to measure the vividness of mental images of risk and the strength of mental representation; words “intense” and “vivid” are synonyms in the Polish language, so we might have assumed that when participants rated images of risk as more intense, they also rated them as more clear and vivid at the same time). Responses to each question were provided on a 10-point scale (the exact wording of these questions and descriptive statistics are shown in Table 1). At the final stage of the experiment, participants were asked to recall risky situations presented in the study to check whether they were similarly involved in the processing of risky situations regardless of the condition (imagery vs. solving arithmetic problems). In this task, five target and five distractor risky situations were displayed on the computer screen in a randomized order, and participants indicated which risky situation was actually shown to them in the study.

**Table 1:** **Descriptive statistics for measures used in Experiment 1**.

**Item**	**Negative**		**Control**	
	**Mean**	***SD***	**Mean**	***SD***
Q1: Does this situation evoke negative emotions? (1—definitely no; 10—definitely yes)	6.08	1.64	4.85	1.94
Q2: Does this situation evoke fear? (1—definitely not; 10—definitely yes)	4.96	1.83	4.27	1.83
Q3: How intense emotions does this situation evoke? (1—very weak; 10—very intense)	5.03	1.68	4.60	1.75
Q4: I think that this situation is …(1—not risky at all; 10—extremely risky)	7.13	1.02	5.66	2.12
Q5: Would you take such risk? (1—definitely not; 10—definitely yes)	4.29	1.65	5.09	1.80
Q6: Rate the intensity of your images of risk (1—not intense at all; 10—extremely intense)	6.02	1.62	4.79	1.99
Negative Affect (Q1, Q2, Q3)	5.36	1.57	4.57	1.69
Risk Perception (Q4, Q5)	5.71	0.80	4.57	0.95
Recall of Risky Situations (percent correct)	0.92	0.24	0.84	0.27
Study duration (in minutes)	13.84	7.42	6.88	2.60

#### Statistical analysis

In each experiment, we fitted a linear mixed model using the lme4 (Bates et al., 2014) and the lmerTest packages implemented in the R statistical environment (R Core Team, 2014). In each model, risk perception was predicted by the experimental manipulation (i.e., images of negative risk consequences vs. control) and measures of evoked emotions. We also treated participants and a risk domain as random-intercept effects, whereas emotions were random-slope effects allowed to vary across participants and different risk domains. To test the indirect effect between the experimental manipulation and risk perception through changes in emotions we constructed lower and upper limits of the 95% confidence interval for the indirect path using the Monte Carlo simulation method (Preacher and Selig, 2012) based on 10,000 random samples. If the confidence interval for the indirect effect did not contain zero, we could conclude that the mediation effect was significant (Hayes, 2013).

### Results

#### Manipulation check

We found that participants in the experimental condition rated their mental images of risky situations as more intense than controls, *b* = 1.23, *p* < 0.001 (see Table 1). Importantly, despite it took more time to complete the whole procedure in the experimental than in the control condition, *t*_(103)_ = −6.478, *p* < 0.001, we did not find differences between conditions in terms of the accuracy of risky situation recall, *t*_(103)_ = −1.636, *p* = 0.105, implying that participants were similarly involved in the processing of risky situations regardless of the condition (imagery vs. solving arithmetic problems).

#### Testing for the indirect effect of negative affect

**Figure 1:**
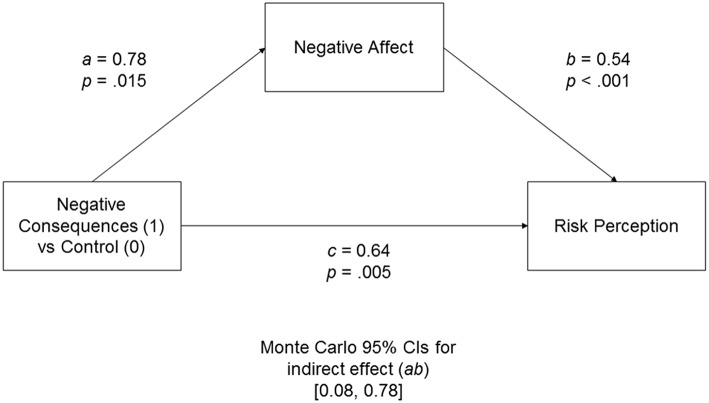
**Unstandardized coefficient for the indirect effect model in Experiment 1 predicting risk perception from negative affect evoked by imagining negative consequences of risk**. Monte Carlo 95% CIs for the indirect effect are based on 10,000 samples.

We averaged three measures of emotions in the way that higher values indicated a more intense negative affect. Similarly, two questions regarding risk were averaged to compute the risk perception measure (i.e., higher values indicated that a situation was perceived as more risky). We found that imagining negative risk consequences (compared to the control condition) increased both negative affect, *a* = 0.78, *p* = 0.015, and risk perception, *c* = 0.64, *p* = 0.005. The indirect path through negative affect was also significant, 95% CIs [0.08, 0.78], indicating that negative affect mediates the relationship between imagining negative risk consequences and risk perception (Figure 1).

### Discussion

Experiment 1 demonstrated that imagining negative consequences of risky activities elicits negative affect that, in turn, leads to higher perceived risk. Importantly, we found that there were no differences between the experimental and control conditions in recalling risky situations which suggests that differences in risk perception were due to mental imagery, and not task involvement (i.e., participants from both conditions were equally motivated and involved in processing risky situations). In this sense, our findings are in line with previous results showing that vividness and intensity of mental images of negative risk consequences determine people's willingness to engage in risk-taking behavior (Traczyk et al., 2015). However, the vital question that arises here is whether the effect is driven solely by mental imagery or rather by negative affect elicited by imagining negative risk consequences. We addressed this question in Experiment 2, in which we modified the procedure used before and asked participants assigned to the control condition to visualize positive risk consequences (instead of solving arithmetic problems). We hypothesized that situations presented to participants would be perceived as more risky because of negative affect elicited by mental images of risk consequences rather than the intensity of mental imagery itself.

## Experiment 2

### Method

#### Participants

One hundred and twenty-four unpaid volunteers (94 females; mean age = 27.1 years; *SD* = 7.3) participated in this study. Participants provided informed consent before the experiment. The study was voluntary, anonymous and in agreement with the guidelines of the Ethical Committee.

#### Procedure

Participants assigned to the experimental condition were asked to read, imagine and write down three negative consequences associated with risk taking in five situations randomly presented on the computer display. Differently than in Experiment 1, participants in the control condition imagined positive risk consequences of five risky situations instead of solving arithmetic problems. Each risky situation was followed by six questions concerning emotions evoked by negative risk consequences (three questions), risk perception (two questions), and the intensity of mental images of risk (one question). Responses were provided on a 10-point scale (the exact wording of these questions and descriptive statistics are presented in Table 2). Similar to Experiment 1, at the end of this experiment participants were also asked to recall risky situations presented in the study.

**Table 2:** **Descriptive statistics for measures used in Experiment 2**.

**Item**	**Negative**		**Positive**	
	**Mean**	***SD***	**Mean**	***SD***
Q1: What kind of emotions does this situation evoke? (1—negative; 10—positive)	3.63	1.88	4.76	2.67
Q2: Does this situation evoke feelings of fear/hope? (1—definitely not; 10—definitely yes)	5.86	2.21	5.67	2.34
Q3: How intense emotions does this situation evoke? (1—very weak; 10—very intense)	5.60	2.61	4.01	2.81
Q4: I think that this situation is …(1—not risky at all; 10—extremely risky)	7.11	2.27	7.19	2.29
Q5: Would you take such risk? (1—definitely not; 10—definitely yes)	4.02	2.65	4.81	2.82
Q6: Rate the intensity of your images of risk (1—not intense at all; 10—extremely intense)	6.07	2.32	5.94	2.35
Negative Affect (Q1, Q2, Q3)	2.74	3.22	0.16	3.40
Risk Perception (Q4, Q5)	6.55	2.15	6.19	2.20
Recall of risky situations (percent correct)	0.98	0.10	0.96	0.14
Study duration (in minutes)	10.04	13.35	15.28	41.37

Participants, when responding to Q3, rated the level of fear in the negative condition and the level of hope in the positive condition. To compute the overall measure of Negative Affect (Q1, Q2, Q3) in the positive condition we inversely coded participants' responses to Q3.

### Results

#### Manipulation check

Intensity of mental images of risk consequences did not differ between negative and positive imagery conditions, *b* = −0.13, *p* = 0.641 (Table 2). Moreover, we did not find any significant differences in the recall of risky situations, *t*_(120)_ = 0.687, *p* = 0.494, nor in time spent on completing the procedure, *t*_(122)_ = −0.949, *p* = 0.344, which suggest that participants were equally involved in processing risky situations regardless of the experimental condition (positive vs. negative risk consequences).

#### Testing for the indirect effect of negative affect

**Figure 2:**
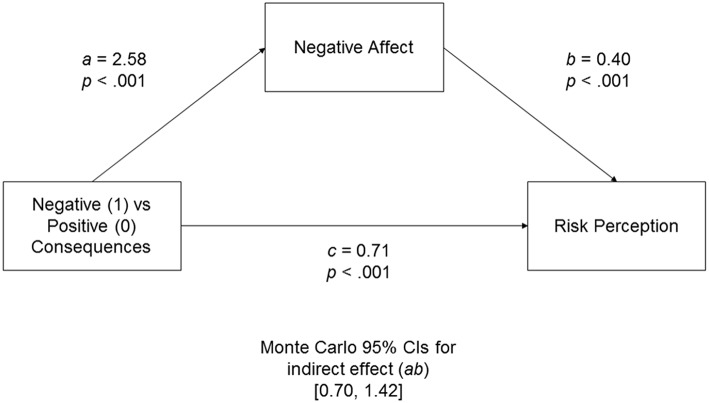
**Unstandardized coefficients for the indirect effect model in Experiment 2 predicting risk perception from negative affect evoked by imagining negative consequences of risk**. Monte Carlo 95% CIs for the indirect effect are based on 10,000 samples.

Similar to Experiment 1, three measures of emotions were averaged in the way that higher values indicated more intense negative affect. Two measures of the perceived risk were used to compute the measure of risk perception (higher values indicated higher perceived riskiness). We found that participants who imagined negative consequences of risk, in comparison to participants imagining positive risk consequences, reported more negative affect, *a* = 2.58, *p* < 0.001, and rated risk as higher, *c* = 0.71, *p* < 0.001. The indirect effect of risk images through negative affect was also significant, 95% CIs [0.70, 1.42], demonstrating that negative images of risk consequences (and not only the intensity of imagery) lead to increased risk perception through negative affect (Figure 2).

### Discussion

Experiment 2 showed that imagining negative, but not positive, consequences of risk influenced risk perception. That is, participants who visualized negative risk consequences rated perceived risk as higher, compared to the control condition, and this relationship was mediated by negative affect. Crucially, we did not find any differences in either the recall of risky situations or in the intensity of mental images of positive or negative risk consequences. These results suggest that it is not the intensity of mental imagery but rather negative affect that plays a key role in risk perception.

However, a question arises of which negative feelings are involved in this process? Is this negative affect or more complex feelings associated with psychological stress? On the basis of several previous studies (Lighthall et al., 2009; Porcelli and Delgado, 2009; Buckert et al., 2014; Traczyk et al., 2015) we argue that elevated perception of risk is likely to be driven by the experienced stress. That is, negative feelings of stress might serve as heuristic information leading to changes in the perceived risk (Slovic et al., 2007). Experiment 3 attempted to verify this hypothesis. We introduced the risk-irrelevant stress manipulation (i.e., stress that is unrelated to a risky situation) and expected that it would lead to higher ratings of perceived risk, compared to the control condition.

## Experiment 3

### Method

#### Participants

Thirty-two undergraduate students (16 females) aged 23.8 years on average (*SD* = 7.2) participated in this study for credit points. None of them had diagnosed arrhythmia or other cardiovascular diseases. Participants were asked to restrain from physical activity, smoking, drinking coffee or energy drinks, and eating large meals for 2 h before the study. The study was voluntary, anonymous, and in agreement with the guidelines of the Ethical Committee.

#### Materials and apparatus

The mental arithmetic task from the Trier Social Stress Test (Kirschbaum et al., 1993) was used to experimentally increase stress level. This task has been proven in earlier research to raise both cardiac and salivary responses as well as self-reported stress measures (Roy et al., 2001; Dickerson and Kemeny, 2004; Gerin, 2011). Participants were presented with a four-digit number (e.g., 1473) and then asked to cumulatively subtract another two-digit number from it (e.g., 17). They were also instructed to perform calculations aloud, as fast as possible and without errors. Additionally, to intensify the manipulation effect, participants were informed that the task would be performed under time constraints and that their answers would be recorded for subsequent video analysis. In the stress-induction condition (i.e., the mental arithmetic task) the subtraction task with six different sets of numbers was used, whereas in the control condition participants had to count backwards from 10 to 1.

The effects of stress manipulation were tested by two measures: (1) systolic and diastolic blood pressure were registered with a digital blood pressure monitor TMA-880 (error +/− 3 mmHg) manufactured by TechMed®, and (2) positive and negative affect were measured with the Positive and Negative Affect Schedule (PANAS; Watson et al., 1988). This scale consists of 20 adjectives describing different feelings and emotions.

#### Procedure

Participants were tested individually in a dimly-lit laboratory room. Ten minutes of a relaxation stage preceded eight measurements of baseline blood pressure taken at 30-s intervals. Then, participants completed the brief PANAS scale in order to assess their current emotional state. A within-subjects manipulation was introduced and balanced in four blocks. Half of participants started the experiment with two blocks of the mental arithmetic task followed by two blocks of the backward counting task. The order of these tasks was reversed in another group of participants. After two blocks of the mental arithmetic task, PANAS was administered again to control for the effects of the experimental manipulation (i.e., stress induction procedure). During each block three blood pressure readings were taken, after which participants imagined consequences of eight or nine risky situations randomly selected from a total number of 34 risky situations covering different risk domains (the unequal number of risky situations in each block was caused by the random selection mechanism, i.e., the need to divide 34 situations into 4 blocks). Each situation was followed by questions regarding stress (one question) and risk perception (two questions). The exact wording of these questions and descriptive statistics are shown in Table 3. The whole procedure lasted ~30 min.

**Table 3:** **Descriptive statistics for measures used in Experiment 3**.

**Item**	**Stress induction**		**Control**	
	**Mean**	***SD***	**Mean**	***SD***
Q1: Do you find this situation stressful? (1—definitely not; 10—definitely yes)	5.84	2.94	5.52	2.95
Q2: I think that this situation is …(1—not risky at all; 10—extremely risky)	7.11	2.67	6.78	2.81
Q3: Would you take such risk? (1—definitely not; 10—definitely yes)	4.73	3.05	4.66	3.16
Risk Perception (Q2, Q3)	6.19	2.49	6.06	2.61

### Results

#### Data reduction

Cardiovascular data were preprocessed in the following steps: (adapted from: Traczyk et al., 2015): (1) twenty measurements of systolic (SY) and diastolic (DY) blood pressure for each of the participants were transformed into Mean Arterial Pressure (MAP = 2/3^*^DY + 1/3^*^SY); (2) the first three measurements from baseline (at the beginning of the study) were removed from further analysis; (3) the remaining 17 measurements were z-scored for each participant; (4) five measurements from baseline were averaged to control for pre-test baseline level of stress; (5) six averaged measurements from the mental arithmetic and six averaged measurements from the control task served as psychophysiological indicators of evoked stress.

#### Manipulation check

**Figure 3:**
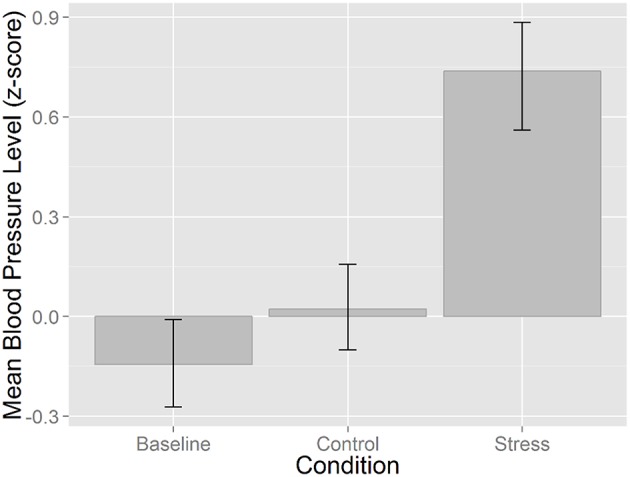
**Mean blood pressure level (*z*-scored) as a function of experimental task (i.e., pre-test baseline readings, control backward counting task, and mental arithmetic task—stress)**. Error bars represent 95% bootstrapped CIs based on 1000 samples.

The transformed blood pressure data was entered into a repeated-measures ANOVA. The main effect of stress manipulation was significant and relatively strong, *F*_(1, 60)_ = 34.095, *p* < 0.001, η^2^ = 0.53. A *post-hoc* analysis with a Bonferroni correction showed enhanced blood pressure in the stress condition compared to both baseline and the control condition (*p*s < 0.001). No difference was found between baseline and the control condition, *p* = 0.366 (Figure 3).

**Figure 4:**
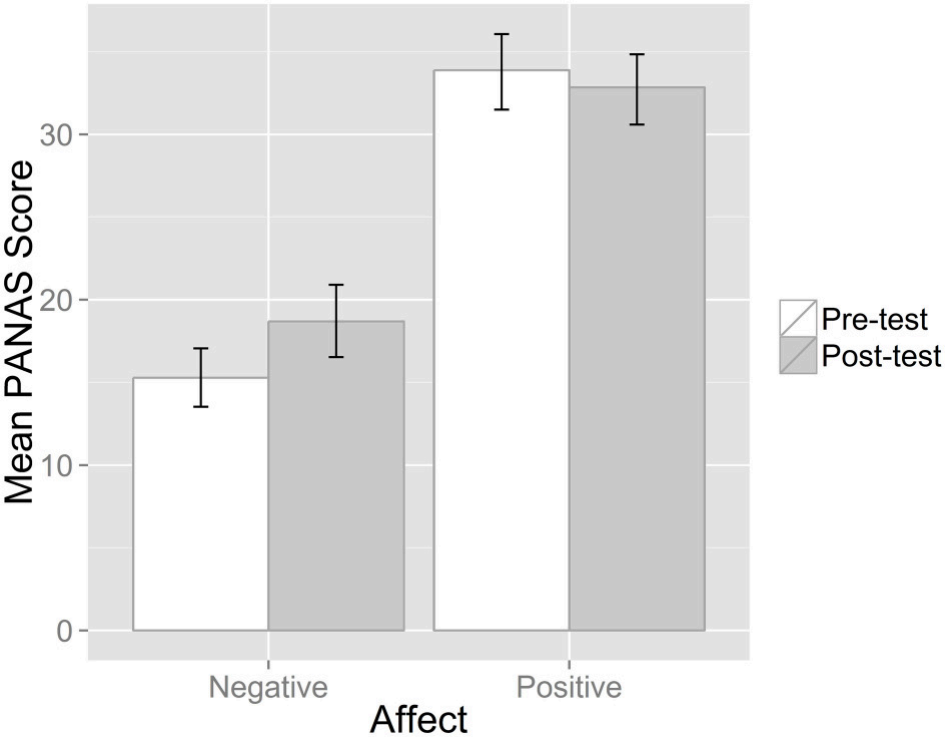
**Mean positive and negative affect measured by PANAS questionnaire before and after stress induction procedure**. Error bars represent 95% bootstrapped CIs based on 1000 samples.

To assess how stress manipulation influenced the positive and negative affect measured with PANAS, two *t*-tests were carried out. Results indicated that experimental manipulation with laboratory-induced stress led to an increase in negative affect, *t*_(31)_ = −3.215, *p* = 0.003. Changes in positive affect were not significant, *t*_(31)_ = 1.187, *p* = 0.224 (Figure 4).

#### Testing for the indirect effect of stress

**Figure 5:**
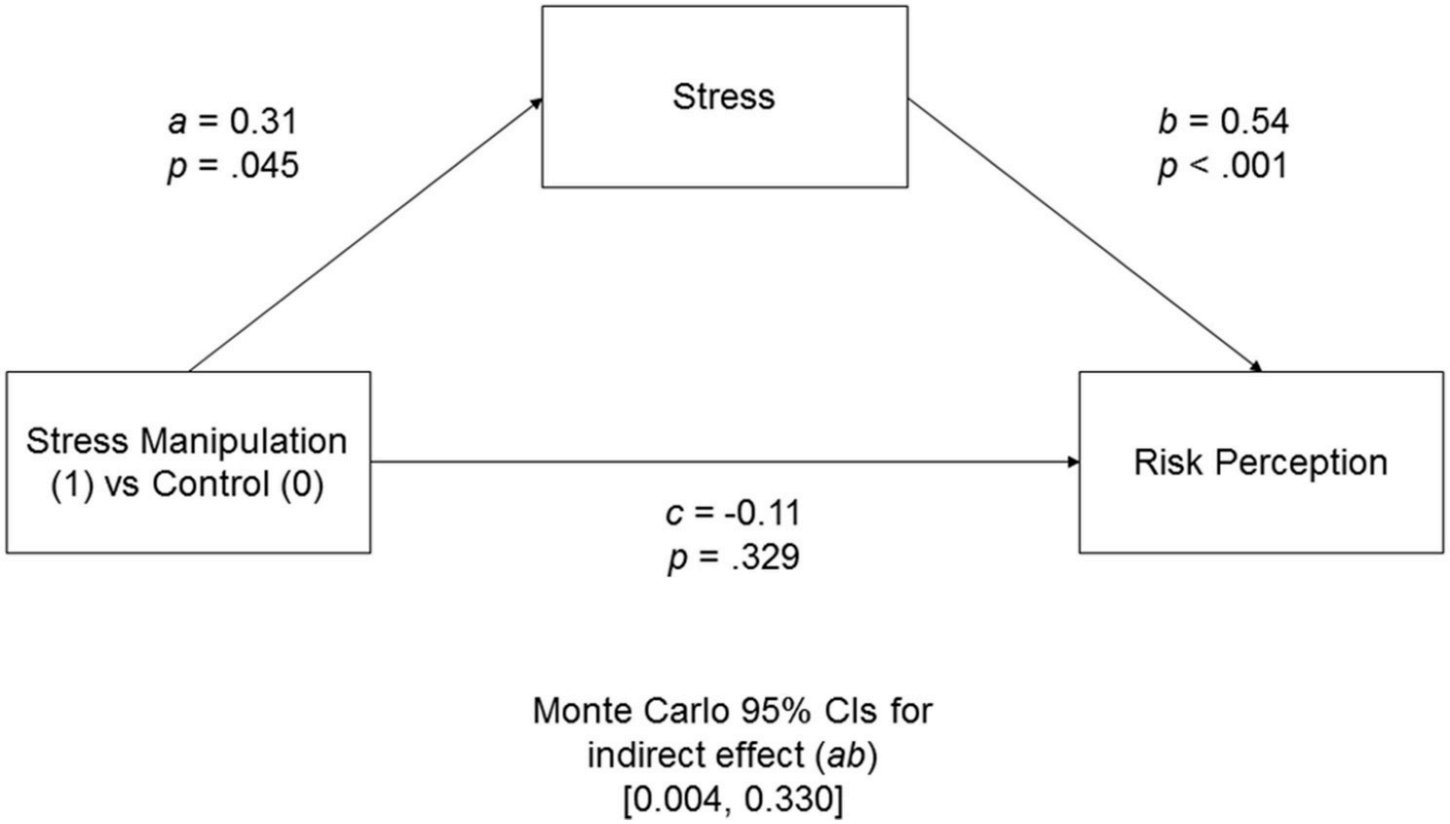
**Unstandardized coefficient for the indirect effect model in Experiment 3 predicting risk perception from risk-irrelevant stress manipulation and ratings of the risky situation's stressfulness**. Monte Carlo 95% CIs for the indirect effect are based on 10,000 samples.

Similar to the two previous experiments, two measures of the perceived risk were used to compute the measure of risk perception (higher values indicated higher perceived riskiness). Next, a mediation analysis, with self-reported stress as a mediator between experimental manipulation and risk perception, was performed (Figure 5). We found that participants who experienced enhanced stress in the experimental condition, compared to the control condition, reported risky situations as more stressful, *a* = 0.31, *p* = 0.045. Stress manipulation, however, did not influence risk perception directly, *c* = −0.11, *p* = 0.329. Crucially, the indirect effect through reported stress level was significant, 95% CIs [0.004, 0.330].

### Discussion

Experiment 3 revealed that risk-irrelevant stress manipulation influenced ratings of the stressfulness of a risky situation, which, in turn, led to higher perceived risk. Moreover, results showing enhanced blood pressure and increased negative affect in response to the experimental manipulation allow us to conclude that changes in risk perception are produced by feelings of stress. These findings lend empirical support for theoretical assumptions in the risk-as-feelings hypothesis (Loewenstein et al., 2001). Specifically, we demonstrated that negative feelings associated with stress might change people's risk perception (i.e., higher stress leads to higher ratings of risk). Crucially, this experiment showed that stress does not have to be directly related to risky activities. Instead, it can be evoked by a completely different task and operate incidentally, influencing risk perception.

## General discussion

A growing body of evidence has been accumulated over the last decade to demonstrate the crucial role of emotions in both risk perception and risk taking (Loewenstein et al., 2001; Loewenstein and Lerner, 2003; Lerner et al., 2004, 2015; Bechara and Damasio, 2005; Slovic et al., 2007). However, much less is known about psychological determinants of people's affective reactions to risk. In this article, we argued that mental images of risk consequences shape affective reactions to risky activities leading to differences in risk perception. Results of three experiments demonstrated that visualizing risk consequences elicits negative affect associated with feelings of stress. Negative affect and stress, in turn, lead to higher perceived risk. Crucially, risk perception is influenced by negative rather than positive affect and this effect is not driven by involvement in processing information about risk.

Our findings provide support for the risk-as-feelings model (Loewenstein et al., 2001), which posits that risk-taking behavior results from feelings, and not only cognitive evaluation. Specifically, we demonstrated that when no information about numerical risk parameters is available (e.g., probability of loss or magnitude of consequences), people rely on mental images of risk consequences. The vividness and intensity of these images produce affective reactions toward risk that change the way it is perceived. However, because participants provided only very brief reports of consequences (see Table S2), we cannot assess the exact content of their mental images of risky situation (e.g., if they are more abstract or concrete). Therefore, basing on these data we are not able to definitely conclude, whether mental representations of risk consequences were visual or prepositional. Nevertheless, studying the content and exact representation of mental images was not the purpose of our research project.

Moreover, the nature of affect investigated in this study is not uniform. Therefore, it seems important to discuss the distinction between the two types of affective influences on risk perception. Firstly, ratings of negative affect that were measured in Experiments 1 and 2 might be interpreted as an example of anticipatory emotions that are integral to the risk perception process. In other words, we assume that when people consider their possible engagement in a risky activity, they anticipate experiencing negative emotions (e.g., fear when imagining a car crash as a consequence of a risky driving). Secondly, we have also shown that risk perception might be influenced by incidental negative affect. Specifically, participants who were under stress unrelated to a risky activity rated perceived risk as higher than participants assigned to the control condition. These findings conform to some extent with the emotion-imbued model (Lerner et al., 2015), which assumes that emotions felt at the time of making the decision are constituted by both feelings integral to a decision problem and incidental emotions that are unrelated to it (Traczyk and Fulawka, 2016).

Despite different sources of affect were examined in this study, we found a consistent pattern of results. Both anticipatory emotions that were integral to a risky activity as well as incidental stress made participants perceive risk as higher. Is it possible that incidental and integral emotions lead to differences in risk perception? Indeed, there is evidence for the emotion-specific influence on risk perception. For example, Lerner and Keltner (2000) reported that fear was associated with higher ratings of risk, whereas anger was related to lower risk ratings. In our study, imagining consequences of risk taking induced fear, but in case of stress manipulation this issue appears to be more complex. The question arises of which negative emotions might have been elicited by the manipulation introducing incidental stress in Experiment 3? For the purpose of this project, we define psychological stress as “a state characterized by strong negative emotions, such as fear, anxiety, anger, hostility, or other emotional states evoking distress, accompanied by physiological and biochemical changes that evidently exceed the baseline level of arousal” (Strelau, 1995, p. 218). Following this definition, in Experiment 3 we observed changes in the stress level, which was operationalized as the increase in negative affect (measured by PANAS) and the increase in the blood pressure level signifying enhanced arousal. Nonetheless, it is less clear whether the task we used to induce stress (i.e., mental arithmetic) elicited fear or rather anger (Moons et al., 2010). Both emotions seem to be plausible. That is, some participants might have experienced fear because they were afraid of making a mistake in calculations, but we cannot exclude that they felt anger because they could not have met the requirements of the task. This problem seems to be worth investigating in future studies. For example, other experimental tasks inducing stress such as the cold pressor task (Lighthall et al., 2009; Porcelli and Delgado, 2009) might be used to test whether the effects of stress on risk perception are independent from the sources of stressful experiences.

In the present project we examined general psychological processes that explain the affective bases of risk perception. However, we also believe that theoretical models that aspire to describe the effects of emotions on risk perception and risk-taking should consider the individual-differences factor as a potential moderator of these relationships. For example, despite numerous studies demonstrating that fearfulness, trait-anxiety, and emotional reactivity moderate the impact of emotions on cognitive processes including decision making (de Visser et al., 2010; Strelau and Zawadzki, 2011; Hartley and Phelps, 2012; Paulus and Yu, 2012; Xu et al., 2013; Matusz et al., 2015), understanding the moderating role of individual differences in the relationship between emotions and risk perception still requires further investigation (for a notable exception see Lerner and Tiedens, 2006).

To summarize, we demonstrated that imagining negative consequences of risk elicits negative affect that leads to higher perceived risk. Importantly, the observed effects are driven by psychological stress that exerts an influence on risk perception even when it is not directly related to a risky activity. Findings of this project suggest that affect-laden imagery producing negative affect and feelings of stress may determine our reactions to risk when no information about numerical risk estimates is provided.

## Author contributions

The authors contributed equally to this work. The experiments were designed, conducted, analyzed and reported by AS, JT, and TZ.

## Funding

The study was supported by Ministry of Science and Higher Education, Poland (BST/WROC/2015/B/6, SWPS University of Social Sciences and Humanities), and the National Science Centre, Poland (2015/17/D/HS6/00703).

### Conflict of interest statement

The authors declare that the research was conducted in the absence of any commercial or financial relationships that could be construed as a potential conflict of interest.
