# Supplementary material for: The Affective Bases of Risk Perception: Negative Feelings and Stress Mediate the Relationship between Mental Imagery and Risk Perception
Source: Front Psychol. 2016 Jun 24;7:932. doi: 10.3389/fpsyg.2016.00932 (PMC4919331; doi:10.3389/fpsyg.2016.00932)
Supplement: Supplementary file 1 [file Table1.DOCX]

Supplementary Material

The affective bases of risk perception: Negative feelings and stress mediate the relationship between mental imagery and risk perception

Agata Sobkow, Jakub Traczyk*, Tomasz Zaleskiewicz

*** Correspondence:** Corresponding Author: jtraczyk@swps.edu.pl

**Table S1**

List of risky situations used in the study. All items were used in Experiment 3. Items used in Experiment 1 and 2 are marked with corresponding superscripts, respectively.

| Speeding |
| --- |
| Parachute jump |
| Hiking alone in wild and unsettled places ^1,2^ |
| Going whitewater rafting |
| Bungee jumping from a high bridge |
| High mountain climbing when the weather is uncertain |
| Engaging in unprotected sex |
| Consuming lots of alcohol during a party |
| Taking drugs |
| Taking strong drugs without consulting your physician ^2^ |
| Ignoring persistent medical problems ^1^ |
| Mixing alcohol with strong medicines |
| Investing large amount of money on a very speculative stock |
| Taking big mortgage loan |
| Investing your money to start a risky business |
| Betting a week’s income on the outcome of a card game |
| Investing 10% of your annual income in bonds |
| Lending a large amount of money to your friend ^1,2^ |
| Defending a controversial issue in a group of unknown people |
| Public correcting your boss/teacher ^1,2^ |
| Public arguing with your boss in the presence of other employees |
| Asking a person you have just met for a date |
| Jumping the long queue |
| Public quitting from saving money for a gift for your colleagues |
| Cheating during an exam ^2^ |
| Passing off a work downloaded from a website as your own |
| Forging signature on an important document |
| Illegally downloading a piece of software or music from Internet |
| Taking a wrong change which was given to you during shopping |
| Spreading negative and untrue information about a person you don’t like ^1^ |
| Playing a gamble in which you have 15% chance to win 900 PLN instead of certain 167 PLN |
| Playing a gamble in which you have 5% chance to win 600 PLN instead of certain 65 PLN |
| Playing a gamble in which you have 95% chance to win 50 PLN instead of certain 38 PLN |
| Playing a gamble in which you have 85% chance to win 250 PLN instead of certain 154 PLN |

**Table S2**

The most frequent negative (Experiment 1 & 2) and positive (Experiment 2) associations with risky situations.

| Situation | The most frequent negative associations (Experiment 1 & 2) | The most frequent positive associations (Experiment 2) |
| --- | --- | --- |
| Public correcting your boss/teacher | firing from a job, poor relationships, vengeance, losing respect and trust, cut salary, reprimand | assertiveness, recognition, respect, being professional, satisfaction, higher self-esteem, showing courage, being proud |
| Ignoring persistent medical problems | death, long stay in hospital, pain, advanced and untreatable stage of disease, disability, expensive treatment, sadness, stress | Not applicable |
| Lending a large amount of money to your friend | losing friend, losing money, debt, quarrel, stress, lack of money for my own purpose, poor relationships, fraud | gratitude, higher self-esteem, feeling helpful, getting trust and respect, satisfaction, better friendship, return a favor in future |
| Hiking alone in wild and unsettled places | death, being lost, loneliness, wild animals, lack of food and water, disease, accident, robbery, anxiety, rape, hypothermia | contact with nature, calm, personal growth and development, meeting new interesting people, adventure, relaxation, freedom, self-efficacy |
| Spreading negative and untrue information about a person you don’t like | losing respect and trust, embarrassment, losing friends, conflict, stress, quarrel, assault, vengeance, court, pricks of conscience, suicide | Not applicable |
| Taking strong drugs without consulting your physician | addiction to drugs, death, side effects, long stay in hospital, overdose, disability, intoxication, pain | fast relief, lack of pain, no queue to physician or specialist, saving time and money, self-efficacy |
| Cheating during an exam | anxiety of being caught red-handed, embarrassment, failing an exam, losing respect, being expelled, resit exam, lack of knowledge, stress | good grade, satisfaction, saving time and effort, adrenaline |
